# Best Possible Approximation Algorithms for Single Machine Scheduling with Increasing Linear Maintenance Durations

**DOI:** 10.1155/2014/547573

**Published:** 2014-02-13

**Authors:** Xuefei Shi, Dehua Xu

**Affiliations:** School of Science, East China Institute of Technology, Nanchang, Jiangxi 330013, China

## Abstract

We consider a single machine scheduling problem with multiple maintenance activities, where the maintenance duration function is of the linear form f(t) = a+bt with a ≥ 0 and b > 1. We propose an approximation algorithm named FFD-LS2I with a worst-case bound of 2 for problem. We also show that there is no polynomial time approximation algorithm with a worst-case bound less than 2 for the problem with b ≥ 0 unless P = NP, which implies that the FFD-LS2I algorithm is the best possible algorithm for the case b > 1 and that the FFD-LS algorithm, which is proposed in the literature, is the best possible algorithm for the case b ≤ 1 both from the worst-case bound point of view.

## 1. Introduction

Scheduling with machine maintenance has attracted many researchers' attention in the last two decades (see, e.g., [1–13]), where high performance approximation algorithms are of great value not only theoretically but also practically.

In a recent paper, Xu et al. [14] study a single machine scheduling problem with multiple maintenance activities which can be formally described as follows. There are *n* independent jobs *J*_1_, *J*_2_,…, *J*_*n*_ to be processed on a single machine. The processing time of job *J*_*i*_ is *p*_*i*_. All jobs are nonpreemptive and are available at time zero. The machine can process at most one job at a time. Assume that the machine has just finished a dummy maintenance activity at time zero. The length of the time interval between any two consecutive maintenance activities is *T* and prefixed. The amount of time needed to perform one maintenance activity is an increasing linear function *T*_*M*_(*t*) = *a* + *bt* of the total processing time *t* of the jobs that are processed after its latest previous maintenance activity, where *a* and *b* are prefixed nonnegative real numbers with *b* ≤ 1. The objective is to schedule all the jobs to the machine such that the makespan, that is, the completion time of the last finished job, is minimized. Extending the well-known three-field *α* | *β* | *γ* classification scheme suggested by Graham et al. [15], the problem is denoted by 1, *MS* [*T*, *T*], *T*_*M*_(*t*) = *a* + *bt*, *b* ≤ 1||*C*_max⁡_ in Xu et al. [14]. For simplicity, in what follows, we denote this problem by *P*_(*b*≤1)_ and its counterpart where *b* > 1 by *P*_(*b*>1)_. Additionally, the problem is denoted by *P*_(*b*>0)_ if *b* > 0.

Xu et al. [14] first propose an approximation algorithm named FFD-LS for the problem *P*_(*b*≤1)_ and then show that its worst-case bound is 2. The underlying idea of their FFD-LS algorithm is straightforward. Viewing the jobs as items, it first packs these items into some bins with the same capacity of *T* by the classical bin-packing algorithm FFD. Let the “jobs” in each used bin plus a maintenance activity whose duration is computed according to the maintenance function *T*_*M*_(*t*) be viewed as a hybrid job. Assign all the hybrid jobs except the last one (i.e., the one with the largest index) to the machine by the classical LS algorithm. Assign the last hybrid jobs to the machine.

For the sake of convenience and completeness, the bin-packing problem, the FFD algorithm, the LS algorithm, and the FFD-LS algorithm are presented as follows.

*Bin-Packing Problem* (see, e.g., Coffman et al. [16]). Given *n* items *a*_1_, *a*_2_,…, *a*_*n*_, each with a size *s*(*a*_*i*_)∈(0, *W*], we are asked to pack them into a minimum number of *W*-capacity bins (i.e., partition them into a minimum number *m* of subsets *B*_1_, *B*_2_,…, *B*_*m*_ such that ∑_*a*_*i*_∈*B*_*j*__ *s*(*a*_*i*_) ≤ *W*, 1 ≤ *j* ≤ *m*).

*Algorithm FFD* (see, e.g., Coffman et al. [16]). Sort all the items such that *s*(*a*_1_) ≥ *s*(*a*_2_) ≥ ⋯≥*s*(*a*_*n*_); for *i* = 1,2,…, *n*, item *a*_*i*_ is packed in the first (lowest-indexed) bin into which it will fit; that is, if there is any partially filled bin *B*_*j*_ with level(*B*_*j*_) ≤ *W* − *s*(*a*_*i*_) where level(*B*_*j*_) is the sum of the sizes of the items that have been packed into bin *B*_*j*_, we place *a*_*i*_ in the lowest-indexed bin having this property. Otherwise, we start a new bin with *a*_*i*_ as its first item.

*Algorithm LS* (see, e.g., Pinedo [17]). Put all the jobs on a list in arbitrary order; then process the jobs consecutively as early as possible.

*Algorithm FFD-LS* (see Xu et al. [14])

*Step* *1*. Construct a bin-packing instance as follows. There are *n* items *a*_1_, *a*_2_,…, *a*_*n*_; the size of item *a*_*i*_ is *p*_*i*_, and the capacity of each bin is *T*. Using the FFD algorithm, assume that we obtain *k* used bins *B*_1_, *B*_2_,…, *B*_*k*_. Let bin *B*_*i*_ be denoted as (*a*_1_^(*i*)^, *a*_2_^(*i*)^,…, *a*_*k*_*i*__^(*i*)^), where *k*_*i*_ is the number of items in *B*_*i*_ and *a*_*j*_^(*i*)^ is the *j*th item assigned to *B*_*i*_.

*Step* *2*. For *i* = 1,2,…, *k*, let *𝒥*_*i*_ = (*J*_1_^(*i*)^, *J*_2_^(*i*)^,…, *J*_*k*_*i*__^(*i*)^, *Ø*_*i*_, *M* ^(*i*)^), where *J*_*j*_^(*i*)^ is the job with a processing time of *p*_*j*_^(*i*)^ corresponding to item *a*_*j*_^(*i*)^, *Ø*_*i*_ is a dummy job with the processing time of *T* − ∑_*j*=1_^*k*_*i*_^ *p*_*j*_^(*i*)^, and *M* ^(*i*)^ denotes a maintenance activity with the length of *a* + level(*B*_*i*_)*b*.

*Step* *3*. Let *𝒥*_*i*_ be viewed as a single job with the processing time of *T* + *a* + level(*B*_*i*_)*b*. Assign *𝒥*_1_, *𝒥*_2_,…, *𝒥*_*k*−1_ to the machine by the LS algorithm. Assign *𝒥*_*k*_ to the machine.

Although the FFD-LS algorithm is originally proposed for *P*_(*b*≤1)_, it can also be applied to *P*_(*b*>1)_ without any modification. However, as can be seen in the next section, there exists an example showing that the worst-case bound of the FFD-LS algorithm can be arbitrarily large for *P*_(*b*>1)_. As a result, we then focus our attention on studying its worst-case bound for *P*_(*b*>1)_ in detail. Specifically, we think of each interval between two consecutive maintenance activities as a batch with a capacity *T* and show that its worst-case bound is 2 for *P*_(*b*>1)_ if the number of nonempty batches in the FFD-LS schedule is not 2. In order to avoid the arbitrarily large worst-case bound, in Section 3, we modify the FFD-LS algorithm and get a new approximation algorithm named FFD-LS2I algorithm with a worst-case bound of 2 for *P*_(*b*>1)_ by adding a step to the FFD-LS algorithm. In Section 4, we study the nonapproximability of the problem and show that there is no polynomial time approximation algorithm with a worst-case bound less than 2 for the problem *P*_(*b*>0)_, which implies that the FFD-LS2I algorithm is the best possible algorithm for *P*_(*b*>1)_ and that the FFD-LS is the best possible algorithm for *P*_(*b*≤1)_ both from the worst-case bound point of view. Finally, in Section 5, we give an answer to another open problem proposed in Xu et al. [14].

## 2. Worst-Case Bound of the FFD-LS Algorithm for *P*_(*b*>1)_

**Example 1**

Consider the scheduling problem *P*_(*b*>1)_ with the following instance: *n* = 2, *T* = *b*, *p*_1_ = *b*, and *p*_2_ = 1. It is easy to see that the FFD-LS algorithm assigns job *J*_1_ to the first batch and *J*_2_ to the second batch and that the corresponding makespan is *C*_max⁡_^FFD-LS^ = *b* + *a* + *b* ^2^ + 1 = *b* ^2^ + *b* + *a* + 1. Note that the optimal schedule assigns job *J*_2_ to the first batch and *J*_1_ to the second batch and that the optimal makespan is *C*_max⁡_* = 3*b* + *a*. Clearly, *C*_max⁡_^FFD-LS^/*C*_max⁡_* → *∞* as *b* → *∞*. In other words, the worst-case bound of the FFD-LS algorithm can be arbitrarily large.

Now, let Λ = ∑_*i*=1_^*n*^ *p*_*i*_. Let S* be an optimal schedule with k* nonempty batches *ℬ*_1_*,…, *ℬ*_k*_* which are indexed according to the processing order. Recall that we assume that there are totally *k* bins being used by the FFD algorithm in the first step of the FFD-LS algorithm. So, for consistency, we may assume that there are totally *k* nonempty batches in the FFD-LS schedule. For convenience, in what follows, these batches are denoted by *ℬ*_1_,…, *ℬ*_*k*_ which are also indexed according to the processing order. Let the total processing times of the jobs in batch *ℬ*_i_* and batch *ℬ*_*i*_ be denoted by *λ*_i_* and *λ*_*i*_, respectively. It is easy to see that the makespan of the optimal schedule is

(1) $$\begin{array}{c} C_{\max}^{*}=\left(k^{*}-1\right)\left(T+a\right)+b\left(\Lambda -\lambda_{k^{*}}^{*}\right)+\lambda_{k^{*}}^{*} \end{array}$$

and that the makespan of the FFD-LS schedule is

(2) $$\begin{array}{c} C_{\max}^{\text{FFD-LS}}=\left(k-1\right)\left(T+a\right)+b\left(\Lambda -\lambda_{k}\right)+\lambda_{k}. \end{array}$$

In what follows, we shall study the worst-case bound of the FFD-LS algorithm for *P*_(*b*>1)_ in detail, which can help us develop a more sophisticated approximation algorithm. Before the study of the worst-case bound, we first present some lemmas.

**Lemma 2**

For the problem *P*_(*b*>1)_, the total processing time of the jobs in any two nonempty batches is strictly larger than *T* in S*.

**Proof**

It suffices to show that *λ*_i_* + *λ*_j_* > *T* for 1 ≤ *i* < *j* ≤ k*.

If there exist two batches, say *ℬ*_i_* and *ℬ*_j_*, such that *λ*_i_* + *λ*_j_* ≤ *T*, where 1 ≤ *i* < *j* ≤ k*, consider the following two cases.

*Case* *1*  (1 ≤ *i* < *j* < k*). Note that *λ*_i_* + *λ*_j_* ≤ *T*. Now, reschedule all the jobs of *ℬ*_i_* into *ℬ*_j_* and delete *ℬ*_i_* with the corresponding maintenance activity. Let the new schedule be denoted by *S*′. It is easy to see that *S*′ is a feasible schedule with a makespan of

(3) $$\begin{array}{c} C_{\max}^{\prime }=\left(k^{*}-2\right)\left(T+a\right)+b\left(\Lambda -\lambda_{k^{*}}^{*}\right)+\lambda_{k^{*}}^{*}. \end{array}$$

Comparing (1) with (3), we have *C*_max⁡_′ < *C*_max⁡_*, which contradicts the optimality of S*.

*Case* *2*  (1 ≤ *i* < k*and j = k*). Similar to Case 1, reschedule all the jobs of *ℬ*_i_* into *ℬ*_j_* and delete *ℬ*_i_* with its corresponding maintenance. Let the new schedule be denoted by *S*′′. It is easy to see that *S*′′ is a feasible schedule with a makespan of

(4) $$\begin{array}{c} C_{\max}^{\prime \prime }=\left(k^{*}-2\right)\left(T+a\right)+b\left(\Lambda -\lambda_{k^{*}}^{*}-\lambda_{i}^{*}\right)+\left(\lambda_{k^{*}}^{*}+\lambda_{i}^{*}\right). \end{array}$$

Recall that *b* > 1. Comparing (1) with (4), we have *C*_max⁡_′′ < *C*_max⁡_*, which again contradicts the optimality of S*.

This completes the proof.

**Lemma 3**

For problem *P*_(*b*>1)_, let *S*′ be a feasible schedule with two nonempty batches and let *S*′′ be a feasible schedule with three nonempty batches. Let the total processing times of the jobs in the first batch of *S*′ and in the second batch of *S*′ be denoted by *λ*_1_′ and *λ*_2_′, respectively. Let the total processing times of the jobs in the first batch of *S*′′, in the second batch of *S*′′, and in the third batch of *S*′′ be denoted by *λ*_1_′′, *λ*_2_′′ and *λ*_3_′′, respectively. If *λ*_1_′ + *λ*_2_′ > *T* and *λ*_*i*_′′ + *λ*_*j*_′′ > *T* for 1 ≤ *i* < *j* ≤ 3, then one has *C*_max⁡_′ < *C*_max⁡_′′, where *C*_max⁡_′ and *C*_max⁡_′′ are the makespans of *S*′ and *S*′′, respectively.

**Proof**

It is easy to see that

(5) $$\begin{array}{c} C_{\max}^{\prime }=T+a+b{\lambda_{1}^{\prime }}_{}+{\lambda_{2}^{\prime }}_{}, \end{array}$$

(6) $$\begin{array}{c} {C_{\max}^{\prime \prime }}_{}=2\left(T+a\right)+b\left({\lambda_{1}^{\prime \prime }}_{}+{\lambda_{2}^{\prime \prime }}_{}\right)+\lambda_{3}^{\prime \prime }. \end{array}$$

Combining (5) with (6), we have

(7) $$\begin{array}{c} {C_{\max}^{\prime }}_{}={C_{\max}^{\prime \prime }}_{}+\left(\lambda_{2}^{\prime }-T\right)-a+b\left(\lambda_{1}^{\prime }-\lambda_{1}^{\prime \prime }-\lambda_{2}^{\prime \prime }\right)-\lambda_{3}^{\prime \prime }. \end{array}$$

Recall that *λ*_1_′′ + *λ*_2_′′ > *T*. So we have

(8) $$\begin{array}{c} C_{\max}^{\prime }<C_{\max}^{\prime \prime }+\left(\lambda_{2}^{\prime }-T\right)-a+b\left(\lambda_{1}^{\prime }-T\right)-\lambda_{3}^{\prime \prime }. \end{array}$$

Note that *λ*_1_′ ≤ *T* and *λ*_2_′ ≤ *T*. So we have

(9) $$\begin{array}{c} C_{\max}^{\prime }<C_{\max}^{\prime \prime }. \end{array}$$

This completes the proof.

**Lemma 4**

*k* ≤ 3k*/2.

**Proof**

View each interval between two consecutive maintenance activities as a bin with capacity *T* and the jobs *J*_*i*_ as items *a*_*i*_ with a size of *p*_*i*_. Let *k*′ be the minimum number of nonempty bins that is needed to pack all the *n* items. It is well known that *k* ≤ 3*k*′/2 (see Simchi-Levi [18]). Note that *k*′ ≤ k*. So we have *k* ≤ 3k*/2. This completes the proof.

**Lemma 5**

For the problem *P*_(*b*>1)_, if *T* < Λ ≤ 2*T*, then 2 ≤ k* ≤ 3 and 2 ≤ *k* ≤ 3.

**Proof**

By *T* < Λ, it is easy to see that 2 ≤ k* and 2 ≤ *k* since that one batch is clearly not sufficient in any feasible schedule. On the other hand, by Lemma 2, we have k* ≤ 3 for otherwise we have Λ > 2*T*, a contradiction. Note that the total processing time of the jobs in any two batches in the FFD-LS schedule is strictly larger than *T*. We thus have *k* ≤ 3. To sum up, we have 2 ≤ k* ≤ 3 and 2 ≤ *k* ≤ 3. This completes the proof.

Now, we are ready to evaluate the worst-case bound of the FFD-LS algorithm in detail.

**Theorem 6**

For the problem *P*_(*b*>1)_, the worst-case bound of the FFD-LS algorithm is not greater than 2 if Λ > 2*T*.

**Proof**

By (1), we have

(10) $$\begin{array}{c} k^{*}=1+\frac{C_{\max}^{*}+\left(b-1\right)\lambda_{k^{*}}^{*}-b\Lambda }{T+a}. \end{array}$$

Note that *k* ≤ 3k*/2 (see Lemma 4). So we have

(11) $$\begin{array}{c} k\leq \frac{3}{2}\left(1+\frac{C_{\max}^{*}+\left(b-1\right)\lambda_{k^{*}}^{*}-b\Lambda }{T+a}\right). \end{array}$$

Substituting (11) into (2), we have

(12) Cmax⁡FFD-LS  ≤32Cmax⁡∗+12(T+a)+32(b−1)λk∗∗−(b−1)λk−b2Λ.

Now, consider the following two cases.

*Case* *1 (*Λ ≥ 3*T).* In this case, we have

(13) $$\begin{array}{c} \frac{b}{2}\Lambda \geq \frac{b}{2}3T>\frac{3}{2}\left(b-1\right)T. \end{array}$$

Substituting (13) into (12), we have

(14) Cmax⁡FFD-LS  ≤32Cmax⁡∗+12(T+a)+32(b−1)(λk∗∗−T)−(b−1)λk.

Note that *λ*_k*_* ≤ *T*, −(*b* − 1)*λ*_*k*_ ≤ 0, and *T* + *a* < *C*_max⁡_*. Combining these three inequalities with (14), we have *C*_max⁡_^FFD-LS^ < 2*C*_max⁡_* and we are done.

*Case* *2 (*2*T* < Λ < 3*T).* In this case, it is easy to see that k* ≥ 3 because two batches are obviously not sufficient in any optimal solution. Note that the total processing time of the jobs in any two nonempty batches is strictly larger than *T* in the FFD-LS schedule. We thus have *k* ≤ 5 for otherwise the total processing times of the jobs will be strictly larger than 3*T*, which violates the assumption of this case.

Combining k* ≥ 3 with (1), we have

(15) $$\begin{array}{c} C_{\max}^{*}\geq 2\left(T+a\right)+b\left(\Lambda -\lambda_{k^{*}}^{*}\right)+\lambda_{k^{*}}^{*}. \end{array}$$

By simple algebra, we have

(16) $$\begin{array}{c} C_{\max}^{*}\geq 2\left(T+a\right)+b\Lambda -\left(b-1\right)\lambda_{k^{*}}^{*}. \end{array}$$

Note that 0 < *λ*_k*_* ≤ *T*. So we have

(17) $$\begin{array}{c} C_{\max}^{*}\geq 2\left(T+a\right)+b\Lambda -\left(b-1\right)T. \end{array}$$

Similarly, combining *k* ≤ 5, 0 < *λ*_*k*_ ≤ *T*, and (2), we have

(18) $$\begin{array}{c} C_{\max}^{\text{FFD-LS}}<4\left(T+a\right)+b\Lambda . \end{array}$$

By simple algebra, (18) becomes

(19) $$\begin{array}{c} C_{\max}^{\text{FFD-LS}}<2\left(2\left(T+a\right)+b\Lambda -\left(b-1\right)T\right)-b\left(\Lambda -2T\right)-2T. \end{array}$$

Substituting (17) into (19), we have

(20) $$\begin{array}{c} C_{\max}^{\text{FFD-LS}}<2C_{\max}^{*}-b\left(\Lambda -2T\right)-2T. \end{array}$$

Recall that 2*T* < Λ; we thus have *C*_max⁡_^FFD-LS^ < 2*C*_max⁡_*.

This completes the proof.

**Theorem 7**

For the problem *P*_(*b*>1)_, the worst-case bound of the FFD-LS algorithm is not greater than 2 if *T* < Λ ≤ 2*T* and *k* = 3.

**Proof**

Substituting *k* = 3 into (2), we have

(21) $$\begin{array}{c} C_{\max}^{\text{FFD-LS}}=2\left(T+a\right)+b\Lambda -\left(b-1\right)\lambda_{3}. \end{array}$$

By Lemma 5, we have 2 ≤ k* ≤ 3. Now, consider the following two cases.

*Case* 1 (k* = 2*).* Substituting k* = 2 into (1), we have

(22) $$\begin{array}{c} C_{\max}^{*}\geq T+a+b\left(\Lambda -\lambda_{2}^{*}\right)+\lambda_{2}^{*}. \end{array}$$

Combining (22) with (21), we have

(23) $$\begin{array}{c} C_{\max}^{\text{FFD-LS}}=2C_{\max}^{*}-\left(b-1\right)\left(\Lambda +\lambda_{3}-2\lambda_{2}^{*}\right)-\Lambda . \end{array}$$

Note that Λ = *λ*_1_ + *λ*_2_ + *λ*_3_. Combining this with (23), we have

(24) Cmax⁡FFD-LS  ≤2Cmax⁡∗−(b−1)((λ1+λ3−λ2∗)+(λ2+λ3−λ2∗))−Λ.

Note that *λ*_1_ + *λ*_3_ > *T* ≥ *λ*_2_* and that *λ*_2_ + *λ*_3_ > *T* ≥ *λ*_2_*. So, by (24), we have *C*_max⁡_^FFD-LS^ ≤ 2*C*_max⁡_*.

*Case* 2 (k* = 3*).* Substituting k* = 3 into (1), we have

(25) $$\begin{array}{c} C_{\max}^{*}=T+a+b\left(\Lambda -\lambda_{3}^{*}\right)+\lambda_{3}^{*}. \end{array}$$

Combining (25) with (21), we have

(26) $$\begin{array}{c} C_{\max}^{\text{FFD-LS}}=C_{\max}^{*}+\left(b-1\right)\left(\lambda_{3}^{*}-\lambda_{3}\right). \end{array}$$

Note that *λ*_3_* − *λ*_3_ ≤ *T* and *b* − 1 ≤ *b*. So we have

(27) $$\begin{array}{c} C_{\max}^{\text{FFD-LS}}\leq C_{\max}^{*}+bT. \end{array}$$

Note that Λ − *λ*_3_* = *λ*_1_* + *λ*_2_* > *T*. Combining this with (25), we have *C*_max⁡_* > *bT*. Substituting this into (27), we have *C*_max⁡_^FFD-LS^ < 2*C*_max⁡_*.

This completes the proof.

**Theorem 8**

For the problem *P*_(*b*>1)_, the worst-case bound of the FFD-LS algorithm is not greater than 2 if *k* ≠ 2.

**Proof**

Consider the following three cases.

*Case* *1 (k* = 1*).* It is trivially obvious that in this case we have *C*_max⁡_^FFD-LS^ = *C*_max⁡_*, and we are done.

*Case* *2 (k* = 3*).* Clearly, in this case, we have Λ > *T*. Now, if *T* < Λ ≤ 2*T*, then, by Theorem 7, we know that the worst-case bound of the FFD-LS algorithm is not greater than 2, and we are done. If 2*T* < Λ, then by Theorem 6, we know that the worst-case bound of the FFD-LS algorithm is not greater than 2 either, and we are also done. This complete the proof of Case 2.

*Case* *3 (k* ≥ 4*).* Clearly, in this case, we have Λ > 2*T*. Again, by Theorem 6, we know that the worst-case bound of the FFD-LS algorithm is not greater than 2, and we are done.

This completes the proof.

## 3. A Modified Approximation Algorithm and Its Worst-Case Bound for *P*_(*b*>1)_

We know from Example 1 and Theorem 8 that the worst-case bound of the FFD-LS algorithm can be arbitrarily large if *k* = 2 and is 2 otherwise. Following the notation in Xu et al. [14], *𝒥*_*i*_ is the hybrid job which consists of the *i*th nonempty bin obtained by the FFD algorithm and the corresponding maintenance activity. Note that *𝒥*_1_ is scheduled before *𝒥*_2_ in the FFD-LS schedule. So we have *λ*_1_ = level(*B*_1_) and *λ*_2_ = level(*B*_2_). In order to avoid the arbitrarily large worst-case bound, as we observe, we only need to add the following step after the FFD-LS algorithm.

*Step* *4*. If *k* = 2 and level(*B*_2_) < level(*B*_1_), interchange *𝒥*_1_ with *𝒥*_2_.

Let us call this modified algorithm FFD-LS2I, which applies algorithm FFD-LS first and then applies Step 4. Clearly, the computational complexity of the FFD-LS2I is *O*(*n* ^2^). Let the FFD-LS2I schedule be denoted by *S* ^⋆^ and let the total processing times of the jobs in the *i*th batch *B*_*i*_^⋆^ of *S* ^⋆^ be denoted by *λ*_*i*_^⋆^. It is easy to see that the number of the nonempty batches in *S* ^⋆^ is also *k* because the fourth step of the FFD-LS2I algorithm does not change the number of batches.

In order to show that the worst-case bound of the FFD-LS2I algorithm is 2, by the structure of the FFD-LS2I algorithm and Theorem 8, we only need to consider the case *k* = 2.

**Theorem 9**

For the problem *P*_(*b*>1)_, the worst-case bound of the FFD-LS2I algorithm is not greater than 2 if *k* = 2.

**Proof**

Clearly, *k* = 2 implies *T* < Λ ≤ 2*T*. Hence, by Lemma 4, we have 2 ≤ k* ≤ 3. Note that the FFD-LS2I schedule has two nonempty batches and that Lemma 3 indicates that the makespan of a feasible schedule with two batches is always less than that of a feasible schedule with three batches. So we have k* = 2.

Substituting k* = 2 into (1), we have

(28) $$\begin{array}{c} C_{\max}^{*}=T+a+b\left(\Lambda -\lambda_{2}^{*}\right)+\lambda_{2}^{*}. \end{array}$$

Similar to (2), the makespan of the FFD-LS2I schedule is

(29) $$\begin{array}{c} C_{\max}^{\text{FFD-LS}2\text{I}}=T+a+b\left(\Lambda -\lambda_{2}^{⋆}\right)+\lambda_{2}^{⋆}. \end{array}$$

Combining these two equations, we have

(30) $$\begin{array}{c} C_{\max}^{\text{FFD-LS}2\text{I}}=2C_{\max}^{*}-T-a-b\left(\Lambda +\lambda_{2}^{⋆}-2\lambda_{2}^{*}\right)+\left(\lambda_{2}^{⋆}-2\lambda_{2}^{*}\right). \end{array}$$

Note that *λ*_1_* + *λ*_2_* = Λ > *T*. We claim that *λ*_2_* > *T*/2 for otherwise we have *λ*_1_* > *T*/2 and thus we can get a schedule with a smaller makespan by interchanging all the jobs in the first batch of S* with those in the second batch of S*, which clearly violates the optimality of S*. Combining this with *λ*_2_^⋆^ ≤ *T*, we have

(31) $$\begin{array}{c} \lambda_{2}^{⋆}-2\lambda_{2}^{*}\leq \lambda_{2}^{⋆}-T\leq 0. \end{array}$$

*Case* *1 (*4*T*/3 ≤ Λ ≤ 2*T).* Note that *λ*_1_^⋆^ ≤ *λ*_2_^⋆^ and that *λ*_1_^⋆^ + *λ*_2_^⋆^ = Λ. So we have *λ*_2_^⋆^ ≥ 2*T*/3. And thus Λ + *λ*_2_^⋆^ − 2*λ*_2_* ≥ 2*T* − 2*λ*_2_*. Combining this with *λ*_2_* ≤ *T*, we have

(32) $$\begin{array}{c} \Lambda +\lambda_{2}^{⋆}-2\lambda_{2}^{*}\geq 0. \end{array}$$

Substituting (31) and (32) into (30), we have

(33) $$\begin{array}{c} C_{\max}^{\text{FFD-LS}2\text{I}}\leq 2C_{\max}^{*}-T-a, \end{array}$$

which implies that *C*_max⁡_^FFD-LS2I^ < 2*C*_max⁡_*.

*Case* *2 (T* < Λ < 4*T*/3*).* Consider the schedule *S* ^⋄^ which schedules the hybrid job *𝒥*_2_ first and then the hybrid job *𝒥*_1_. Let the total processing time of the jobs in the first batch of *S* ^⋄^ and in the second batch of *S* ^⋄^ be denoted by *λ*_1_^⋄^ and *λ*_2_^⋄^, respectively. Clearly, *λ*_1_^⋄^ + *λ*_2_^⋄^ = Λ. Note that Step 4 schedules the hybrid job with the highest level last. So we have *λ*_2_^⋄^ ≤ *λ*_2_^⋆^.

Let the makespan of the schedule *S* ^⋄^ be denoted by *C*_max⁡_^⋄^. It is easy to see that

(34) $$\begin{array}{c} C_{\max}^{⋄}=T+a+b\left(\Lambda -\lambda_{2}^{⋄}\right)+\lambda_{2}^{⋄}. \end{array}$$

Recall that *λ*_2_^⋄^ ≤ *λ*_2_^⋆^ and *b* > 1. Comparing (29) with (34), we have

(35) $$\begin{array}{c} C_{\max}^{\text{FFD-LS}2\text{I}}\leq C_{\max}^{⋄}. \end{array}$$

Now, consider two subcases.

*Subcase 2.1*. There is only one job, say job *J*_*u*_, in the first batch of the schedule *S* ^⋄^. Without loss of generality, we assume that the jobs in *𝒥*_1_ are ordered in nondecreasing order of their processing times; that is, *p*_1_^(1)^ ≥ *p*_2_^(1)^ ≥ ⋯≥*p*_*k*_1__^(1)^. Suppose that *p*_*s*_^(1)^ ≥ *p*_*u*_ > *p*_*s*+1_^(1)^. Clearly, we have

(36) $$\begin{array}{c} \sum_{i=1}^{s}p_{i}^{\left(1\right)}+p_{u}>T, \end{array}$$

(37) $$\begin{array}{c} \lambda_{2}^{⋄}=\Lambda -p_{u}. \end{array}$$

Now, consider the position of job *J*_*u*_ in the optimal schedule S*. If *J*_*u*_ is in the first batch of S*, then we have *λ*_2_* ≤ Λ − *p*_*u*_. Combine this with (37), we have *λ*_2_^⋄^ ≥ *λ*_2_*. If *J*_*u*_ is in the second batch of S*, then at least one job, say *J*_*v*_^(1)^ with 1 ≤ *v* ≤ *s*, must be assigned to the first batch of S*. To see this is the case, suppose all the jobs *J*_1_^(1)^,…, *J*_*s*_^(1)^ are assigned to the second batch of S*. We thus have *λ*_2_* ≥ ∑_*i*=1_^*s*^ *p*_*i*_^(1)^ + *p*_*u*_. Combining this with (36), we have *λ*_2_* > *T*, which implies that S* is infeasible, clearly, a contradiction. Now, suppose *J*_*v*_^(1)^ is in the first batch of S*, where 1 ≤ *v* ≤ *s*. Note that *p*_*v*_^(1)^ ≥ *p*_*u*_. So we have *λ*_2_* ≤ Λ − *p*_*v*_^(1)^ ≤ Λ − *p*_*u*_. Combining this with (37), we have *λ*_2_^⋄^ ≥ *λ*_2_*. So, in either case, we have *λ*_2_^⋄^ ≥ *λ*_2_*. Combining this with (28) and (34), we have *C*_max⁡_^⋄^ ≤ *C*_max⁡_*. Combining this with (35), we have *C*_max⁡_^FFD-LS2I^ ≤ *C*_max⁡_*, which implies that *C*_max⁡_^FFD-LS2I^ = *C*_max⁡_* by the optimality of S*.

*Subcase 2.2*. There are at least two jobs in the first batch of the schedule *S* ^⋄^. Let *J*_*l*_ be the job with the smallest processing time in the first batch of the schedule *S* ^⋄^. Clearly, we have

(38) $$\begin{array}{c} \lambda_{1}^{⋄}\geq 2p_{l},\\ \lambda_{2}^{⋄}+p_{l}>T\geq \lambda_{2}^{*}. \end{array}$$

Combining (28) with (34), we have

(39) $$\begin{array}{c} C_{\max}^{⋄}=2C_{\max}^{*}-T-a-b\left(\Lambda +\lambda_{2}^{⋄}-2\lambda_{2}^{*}\right)+\left(\lambda_{2}^{⋄}-2\lambda_{2}^{*}\right). \end{array}$$

By (38), we know that

(40) $$\begin{array}{c} \Lambda +\lambda_{2}^{⋄}-2\lambda_{2}^{*}\geq \Lambda -\lambda_{2}^{⋄}-2p_{l}. \end{array}$$

Recall that Λ − *λ*_2_^⋄^ = *λ*_1_^⋄^ and that *λ*_1_^⋄^ ≥ 2*p*_*l*_. So we have

(41) $$\begin{array}{c} \Lambda +\lambda_{2}^{⋄}-2\lambda_{2}^{*}\geq 0. \end{array}$$

By a similar reasoning as in the arguments of deducing (31), we have

(42) $$\begin{array}{c} \lambda_{2}^{⋄}-2\lambda_{2}^{*}\leq \lambda_{2}^{⋄}-T\leq 0. \end{array}$$

Substituting (41) and (42) into (39), we have

(43) $$\begin{array}{c} C_{\max}^{⋄}\leq 2C_{\max}^{*}-T-a, \end{array}$$

which implies that *C*_max⁡_^⋄^ ≤ 2*C*_max⁡_*. Combining this with (35), we have *C*_max⁡_^FFD-LS2I^ ≤ 2*C*_max⁡_*.

This completes the proof.

Now, we are ready to present the worst-case bound of the FFD-LS2I algorithm.

**Theorem 10**

For the problem *P*_(*b*>1)_, the worst-case bound of the FFD-LS2I algorithm is 2 and the bound is tight.

**Proof**

If *k* ≠ 2, it is clear that the FFD-LS2I algorithm is reduced to the FFD-LS algorithm, and thus, by Theorem 8, we know that the worst-case bound of the FFD-LS2I algorithm is not greater than 2. If *k* = 2, then, by Theorem 9, we know that the worst-case bound of the FFD-LS2I algorithm is not greater than 2 either. Hence, we have completed the proof that the worst-case bound of the FFD-LS2I algorithm is not greater than 2.

To show that this bound cannot be smaller than 2, consider the following instance. Let *T* = 12, *p*_1_ = 8 − 2/*b*, *p*_2_ = *p*_3_ = 4 + 1/*b*, *p*_4_ = 4, *p*_5_ = 4 − 2/*b*, *p*_6_ = 2/*b*, and *a* = 1. It is easy to see that *C*_max⁡_^FFD-LS2I^ = 24 + 24*b* + 2/*b* while *C*_max⁡_* = 25 + 12*b*. It follows that *C*_max⁡_^FFD-LS2I^/*C*_max⁡_* = (24 + 24*b*)/(25 + 12*b*) → 2 as *b* → *∞*.

This completes the proof.

## 4. Nonapproximability

In this section, we shall show that it is impossible to have a polynomial time approximation algorithm with a worst-case bound of less than 2 for the scheduling problem *P*_(*b*≥0)_ unless *P* = NP, which implies that the proposed algorithm FFD-LS2I is the best possible approximation algorithm for *P*_(*b*>1)_ from the worst-case bound point of view and that the FFD-LS algorithm proposed by Xu et al. [14] is the best possible approximation algorithm for *P*_(*b*≤1)_ from the same point of view.

Note that the case *b* = 0 has been considered in Ji et al. [3] (see the following lemma, that is, Lemma 11), so we only need to consider the case *b* > 0, that is, the problem *P*_(*b*>0)_.

**Lemma 11 (Ji et al. [3])**

There is no polynomial time approximation algorithm with a worst-case bound less than 2 for the scheduling problem *P*_(*b*=0)_ unless *P* = NP.

The underlying idea of our approach for problem *P*_(*b*>0)_ is by contradiction. Specifically, we shall show that if there exists an approximation algorithm with a worst-case bound of 2 − *ϵ* with 0 < *ϵ* < 1 for *P*_(*b*>0)_ then it can be used to establish a polynomial time algorithm for the well-known NP-complete problem Partition. This clearly leads to a contradiction if *P*≠NP. Hence, such an approximation algorithm for the scheduling problem *P*_(*b*>0)_ cannot exist unless *P* = NP.

*Partition Problem* (see, e.g., Garey and Johnson [19]). Given *n* positive integers *h*_1_, *h*_2_,…, *h*_*n*_ with ∑_*i*=1_^*n*^ *h*_*i*_ = 2*H*, does there exist a set *X*⊆{1, 2,…, *n*} with ∑_*i*∈*X*_ *h*_*i*_ = *H*?

For any fixed positive number *ϵ* < 1 and an instance I of the Partition problem, we construct an instance II of the scheduling problem *P*_(*b*>0)_ as follows. There are *n* jobs *J*_1_, *J*_2_,…, *J*_*n*_ and the processing time of job *J*_*i*_ is *p*_*i*_ = *h*_*i*_. Let *T* = *H* and *a* = (2*T* + *bT*)(1 − *ϵ*)/*ϵ* + 1. It is clear that this construction can be performed in polynomial time.

**Theorem 12**

If there exists a polynomial time approximation algorithm *A*_*ϵ*_ with a worst-case bound of 2 − *ϵ* for some positive number *ϵ* < 1 for the scheduling problem *P*_(*b*>0)_, then there exists a polynomial time algorithm for the Partition problem.

**Proof**

Given any instance I of the Partition problem, we construct the corresponding instance II of the scheduling problem *P*_(*b*>0)_ in polynomial time as stated above. Let *C*_max⁡_^*A*_*ϵ*_^ and *C*_max⁡_* be the makespan of the schedule produced by the approximation algorithm *A*_*ϵ*_ and the makespan of an optimal schedule S* for the instance II of *P*_(*b*>0)_, respectively. We shall show that the instance I of the Partition problem can be answered by merely comparing the values of *C*_max⁡_^*A*_*ϵ*_^ and 2(*T* + *a*) + *bT*.

To see that this is the case, let us apply algorithm *A*_*ϵ*_ to instance II. We claim that if *C*_max⁡_^*A*_*ϵ*_^ ≤ 2(*T* + *a*) + *bT* then there exists a solution to instance I of the Partition problem and, otherwise, there does not exist a solution to instance I. In what follows, we shall show that this claim is correct.

*Case* *1*  (*C*_max⁡_^*A*_*ϵ*_^ ≤ 2(*T* + *a*) + *b* *T).* Clearly, we have *C*_max⁡_* ≤ 2(*T* + *a*) + *bT* because *C*_max⁡_* ≤ *C*_max⁡_^*A*_*ϵ*_^. Let k* be the total number of batches used in S*. Note that *T* = *H* and Λ = ∑_*i*=1_^*n*^ *p*_*i*_ = 2*H*, so we have k* ≥ 2.

Let *λ*_i_* be the total processing time of the jobs in the *i*th batch of S*. If k* ≥ 3, then, by (1), we have *C*_max⁡_* ≥ 2(*T* + *a*) + *b*(Λ − *λ*_k*_*) + *λ*_k*_*. Note that Λ − *λ*_k*_* = ∑_*i*=1_^k*−1^ *λ*_i_* ≥ *λ*_1_* + *λ*_2_* > *T*. So we have *C*_max⁡_* > 2(*T* + *a*) + *bT*, which contradicts the inequality *C*_max⁡_* ≤ 2(*T* + *a*) + *bT*. This implies that k* = 2. That is, the optimal schedule S* has two nonempty batches. Now, let *X* be the set of indices of the jobs scheduled in the first batch of the optimal schedules. Clearly we have ∑_*i*∈*X*_ *p*_*i*_ = *T*. Recall that *T* = *H* and *p*_*i*_ = *h*_*i*_ for *i* = 1,2,…, *n*. So we have ∑_*i*∈*X*_ *h*_*i*_ = *H*, which implies that there exists a solution to the instance I of the Partition problem.

*Case* *2 (C*_max⁡_^*A*_*ϵ*_^ > 2(*T* + *a*) + *b* *T).* Recall that the worst-case bound of the algorithm *A*_*ϵ*_ is 2 − *ϵ*. So we have

(44) $$\begin{array}{c} C_{\max}^{*}\geq \frac{C_{A_{\epsilon }}}{2-\epsilon }>\frac{2\left(T+a\right)+bT}{2-\epsilon }. \end{array}$$

Recall that *a* = (2*T* + *bT*)(1 − *ϵ*)/*ϵ* + 1. So we have

(45) $$\begin{array}{c} \epsilon =\frac{2T+bT}{2T+bT+a-1}. \end{array}$$

Substituting (45) into (44), we have

(46) Cmax⁡∗>2(T+a)+bT2−(2T+bT)/(2T+bT+a−1)=2T+bT+a+2T+bT2T+bT+2a−2≥2T+bT+a.

If there exists a solution, namely, set *X*, to the instance I of the Partition problem, let the jobs whose indices belong to the set *X* be scheduled into the first batch and the remaining jobs the second batch; we then obtain a schedule with a makespan of 2*T* + *a* + *bT*, which clearly contradicts (46). Hence, there does not exist a solution to the instance I of the Partition problem.

This completes the proof.

By Theorem 10 and the fact that no NP-complete problem can be solved by a polynomial time algorithm unless *P* = NP, we have the following result.

**Theorem 13**

There is no polynomial time approximation algorithm with a worst-case bound less than 2 for the scheduling problem *P*_(*b*>0)_ unless *P* = *NP*.

Combining Lemma 11 and Theorem 13, we have the following result.

**Theorem 14**

There is no polynomial time approximation algorithm with a worst-case bound less than 2 for the scheduling problem *P*_(*b*≥0)_ unless *P* = *NP*.

Recall that we have showed in Theorem 10 that the worst-case bound of the FFD-LS2I algorithm is 2 for *P*_(*b*>1)_. Combining this with Theorem 14, we have the following result.

**Theorem 15**

The FFD-LS2I algorithm is the best possible polynomial time approximation algorithm for *P*_(*b*>1)_ unless *P* = *NP* from the worst-case bound point of view.

Recall that Xu et al. [14] have showed that the worst-case bound of the FFD-LS algorithm is 2 for *P*_(*b*≤1)_. Combining this with Theorem 14, we have the following result.

**Theorem 16**

The FFD-LS algorithm is the best possible polynomial time approximation algorithm for *P*_(*b*≤1)_ unless *P* = *NP* from the worst-case bound point of view.

## 5. An Answer to Another Open Problem

Viewing each batch as a bin, in Remark 3 of Section 4 of the paper of Xu et al. [14], Xu et al. claim that “it seems that it is not necessary for an optimal schedule of 1, *MS*[*T*, *T*], *T*_*M*_(*t*) = *a* + *bt*, *b* > 2 + *a*/*T*||*C*_max⁡_ to have the minimum number of bins. Whether this is true may be an interesting problem for further study.” The following example gives an answer to this open problem.

**Example 17**

Consider the following instance for the scheduling problem under consideration: *T* = 20, *a* = 1, *b* = 12, *n* = 8, *p*_1_ = *p*_2_ = *p*_3_ = *p*_4_ = 13, and *p*_5_ = *p*_6_ = *p*_7_ = *p*_8_ = 5. It is easy to see that the minimum number of bins for this instance is 4; see, for example, that job *J*_*i*_ and job *J*_*i*+4_ are packed into bin *B*_*i*_ for *i* = 1,…, 4. The corresponding makespan is 729. However, if we assign job *J*_*i*_ to the *i*th bin for *i* = 1,…, 4 and assign the remaining jobs to the fifth bin, we get an optimal schedule with a makespan of 728. Clearly, this implies that it is not necessary for an optimal schedule of the considered scheduling problem to have the minimum number of bins.

## 6. Conclusion

In this paper, we revisited the single machine scheduling problem considered in Xu et al. [14]; we provided an approximation named FFD-LS2I with a worst-case bound of 2 for the case *b* > 1. We showed that the FFD-LS2I algorithm is the best possible algorithm for the case *b* > 1 and that the FFD-LS algorithm is the best possible algorithm for the case *b* ≤ 1 both from the worst-case bound point of view. We also give an answer to another open problem proposed in Xu et al. [14].

Future research may focus on the design of approximation algorithms with a lower time complexity while maintaining the worst-case bound of 2. The other maintenance duration functions such as convex function and concave function are also worth considering.
